# Long-distance transport of *Gibberellic Acid Insensitive* mRNA in *Nicotiana benthamiana*

**DOI:** 10.1186/1471-2229-13-165

**Published:** 2013-10-21

**Authors:** Haiyan Xu, Reika Iwashiro, Tianzhong Li, Takeo Harada

**Affiliations:** 1 Faculty of Agriculture and Life Science, Hirosaki University, Hirosaki 036-8561, Japan; 2 The United Graduate School of Agricultural Sciences, Iwate University, Morioka 020-8550, Japan; 3 Laboratory of Fruit Cell and Molecular Breeding, China Agriculture University, Beijing 100193, China; 4 Present address: Research Institute of Forestry, Chinese Academy of Forestry, Beijing 100091, China

**Keywords:** *GAI*, mRNA, Transport, Grafting, Dwarf, Phloem

## Abstract

**Background:**

The Gibberellic Acid (GA) signal is governed by the *GAI* (*Gibberellic Acid Insensitive*) repressor, which is characterized by a highly conserved N-terminal DELLA domain. Deletion of the DELLA domain results in constitutive suppression of GA signaling. As the *GAI* transcript is transportable in phloem elements, a Δ-DELLA *GAI* (*gai*) transgenic stock plant can reduce the stature of a scion through transport of *gai* mRNA from the stock. However, little is known about the characteristics of a scion on a *gai* stock.

**Results:**

Arabidopsis Δ-DELLA *GAI* (*gai*) was fused with a T7 epitope tag and expressed under the control of a companion cell-specific expression promoter, *Commelina yellow mottle virus* promoter (CoYMVp), to enhance transport in the phloem. The CoYMVp:Atgai-T7 (*CgT*) transgenic *Nicotiana benthamiana* exhibited a dwarf phenotype and lower sensitivity to GA enhancement of shoot stature. A wild-type (WT) scion on a *CgT* stock contained both *Atgai-T7* mRNA and the translated product. Microarray analysis to clarify the effect of the *CgT* stock on the gene expression pattern in the scion clearly revealed that the WT scions on *CgT* stocks had fewer genes whose expression was altered in response to GA treatment. An apple rootstock variety, *Malus prunifolia*, integrating CoYMVp:Atgai moderately reduced the tree height of the apple cultivar scion.

**Conclusions:**

Our results demonstrate that *Atgai* mRNA can move from companion cells to sieve tubes and that the translated product remains at the sites to which it is transported, resulting in attenuation of GA responses by reducing the expression of many genes. The induction of semi-dwarfism in an apple cultivar on root stock harbouring *Atgai* suggests that long-distance transport of mRNA from grafts would be applicable to horticulture crops.

## Background

The importance of gibberellins (GAs) to angiosperm growth regulation has been demonstrated by the phenotype of GA-deficient mutants. The GA-deficient *Arabidopsis thaliana ga1-3* mutant that lacks *ent*-kaurene synthetase A, an enzyme in the GA biosynthesis pathway, exhibits a characteristic severe dwarf phenotype [1]. Mutants such as *ga1-3* are GA-sensitive dwarf mutants that have been observed in a number of different plant species and typically carry recessive mutations that reduce the activity of GA biosynthesis enzymes [2]. Further molecular characterization of various GA response mutants led to the discovery of the *GID1* (*GIBBERELLIC INSENSITIVE DWARF1*) and DELLA proteins, which are key components of the molecular GA-GID1-DELLA mechanism that enables plants to respond to GA [3]. Genetic and molecular studies have identified the GA receptors and several positive and negative components of the GA signaling cascade [4,5]. Among them, the three major players are the GA receptors, the DELLA repressor proteins, and the F-box proteins that control the stability of DELLA proteins. Ueguchi-Tanaka et al. [6] demonstrated that *GID1* is a soluble GA receptor in rice (*Oryza sativa*). Discovery of the molecular identity of the endogenous plant GA-opposable growth-inhibitory factor resulted from molecular cloning of the genes encoding what are now known as the DELLA proteins.

The *Arabidopsis gai* mutation confers dominant, GA-insensitive dwarfism [7,8]. An insertional mutagenesis approach has facilitated the molecular cloning of *gai* via isolation of a *Ds* transposon-inactivated allele [9]. The *gai* open reading frame carries a small in-frame deletion mutation and thus encodes an altered product, a mutant gai protein that lacks a 17-amino-acid segment, now known as the DELLA domain, named after its first five amino acids. Molecular genetic analysis of GA-insensitive dwarf mutants has also identified an F-box protein (SLY1) that is part of a DELLA-interacting E3 ubiquitin ligase that interacts with a C-terminal region of the DELLA protein [10-12] and targets DELLAs for breakdown by the proteasome. DELLA proteins are thought to repress plant growth, and gibberellins promote growth by overcoming the repressive effects of these proteins. The *Arabidopsis gai-1* mutant has a 51-bp deletion that encodes part of the conserved DELLA domain. As mentioned above, the Δ-DELLA form of GAI acts as a gain-of-function mutant that can inhibit some components of the GA signaling pathway [9]. Expression of *Arabidopsis gai* in rice yields a dwarf phenotype, suggesting that GAI is sufficiently conserved between plant families to allow it to function [13].

Haywood et al. [14] have reported the long-distance delivery of RNA for the *Arabidopsis ΔDELLA-gai* (*Atgai*) genes. In grafting experiments, they demonstrated that the *gai* transcript specifically entered functional sieve elements and induced a highly reproducible change of leaf phenotype in tomato when *Atgai* mRNA was transported into the tomato shoot apex [14]. Long-distance transport of the GAI transcript in woody plants (*Malus* and *Pyrus*) has also been demonstrated [15,16]. Ham et al. [17] reported that the polypyrimidine tract binding motif within the *GAI* mRNA is involved in the formation of a mobile ribonucleoprotein complex, and proposed the presence of motifs that are necessary and sufficient for long-distance trafficking of the *GAI* transcript. Furthermore, Huang and Yu [18] reported that the trafficking of GAI RNA is mediated by specific RNA motifs existing among coding sequences and the 3′-untranslated regions.

Experiments using a grafting system have provided long-distance transport of several transcripts across a graft union, such as *CmPP16* (encoding a 16-kDa *Cucurbita maxima* phloem protein) [19], *CmNACP* (*Cucurbita maxima* non-cell-autonomous protein) [20], *PFP* (pyrophosphate-dependent phosphofructokinase) *-LeT6*[21,22], *StBEL5* (*Solanum tuberosum* BEL1-like transcription factor) [23,24] and *AUX/IAA14*[25,26]. However, no details of the molecular mechanism involved, especially the physiological function of the *GAI* mRNA transport system, have been clarified. In the present study, we characterized *GAI* mRNA transport through phloem using *Atgai* transgenic tobacco as the experimental material. The results proved that a WT scion on *Atgai* rootstock contained the Atgai protein and that its growth reflected attenuation of the expression of many GA response genes.

## Results

### *Atgai* transgenic tobacco exhibits dwarfism and lower sensitive to GA_3_

**Figure 1:**
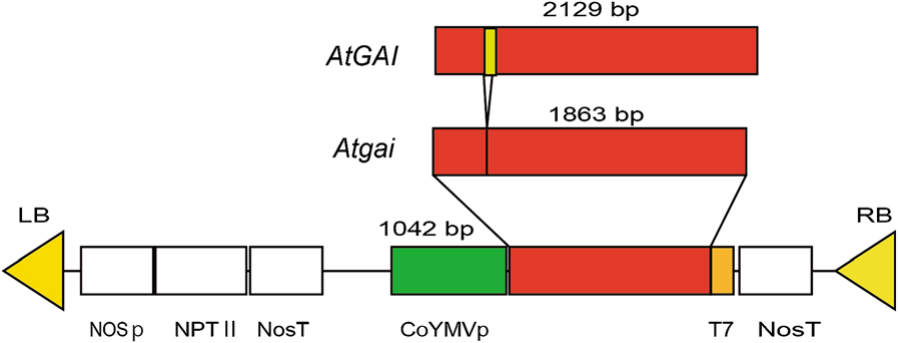
**Vector structure of *CgT*.***CoYMVp; Commelina yellow mottle virus* promoter; *Atgai*: *Arabidopsis thaliana gai* gene (a gain-of-function DELLA allele of *AtGAI*), T7; T7-epitope tag (an 11-amino-acid peptide encoded in the leader sequence of T7 bacteriophage gene 10), Np; Nos promoter, NPT II; A gene encoding kanamycin resistance (primarily neomycin phosphotransferase II), Nt; Nos terminator.

**Figure 2:**
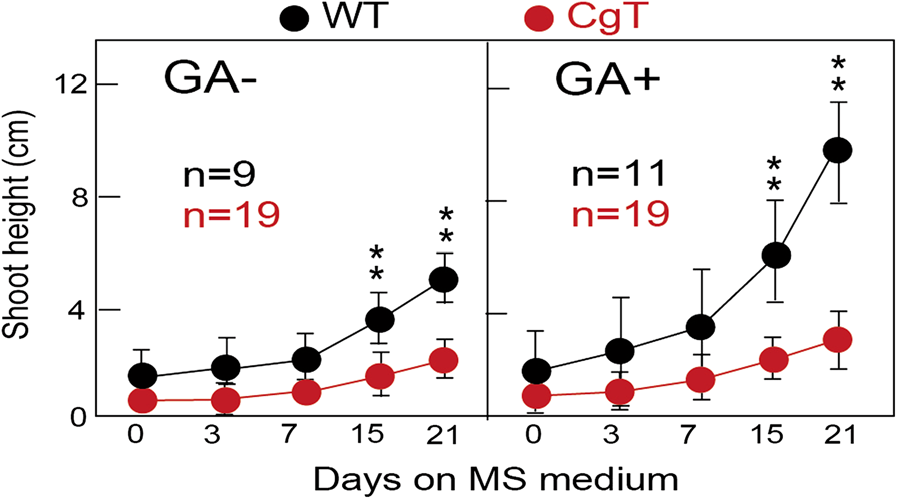
**Growth rates of *CgT *and WT.***CgT* and WT tobacco were grown on MS medium in a bottle for three weeks. GA+ indicates the results obtained from plants grown on MS agar containing GA_3_. **P < 0.01 (Student’s *t* test) for comparisons between *CgT* and WT.

Transgenic plants over expressing *Atgai* and showing a dwarf phenotype have been demonstrated in tomato, tobacco and apple [16,27,28]. Since *Atgai* mRNA has been shown to be transportable through phloem, a T-DNA construct harboring a construct expressing *Atgai* (*CgT*) driven by a companion cell-specific promoter (Figure 1) was integrated into *N. benthamiana* by *Agrobacterium* transformation. *CgT* transgenic tobacco plants clearly exhibited a semi-dwarf phenotype and did not show accelerated growth from 7 days after planting, as was the case for WT plants. Even after GA treatment, the *CgT* plants showed only a small increase in stature, being about one fourth that of WT plants (Figure 2).

### *CgT* rootstock affects WT scion growth

**Figure 3:**
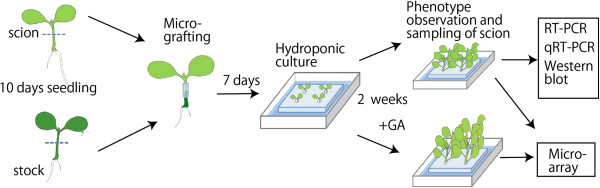
**Illustration of the experimental procedure using grafted tobacco plants.** Respective methodological details appear in each section of the body text.

**Figure 4:**
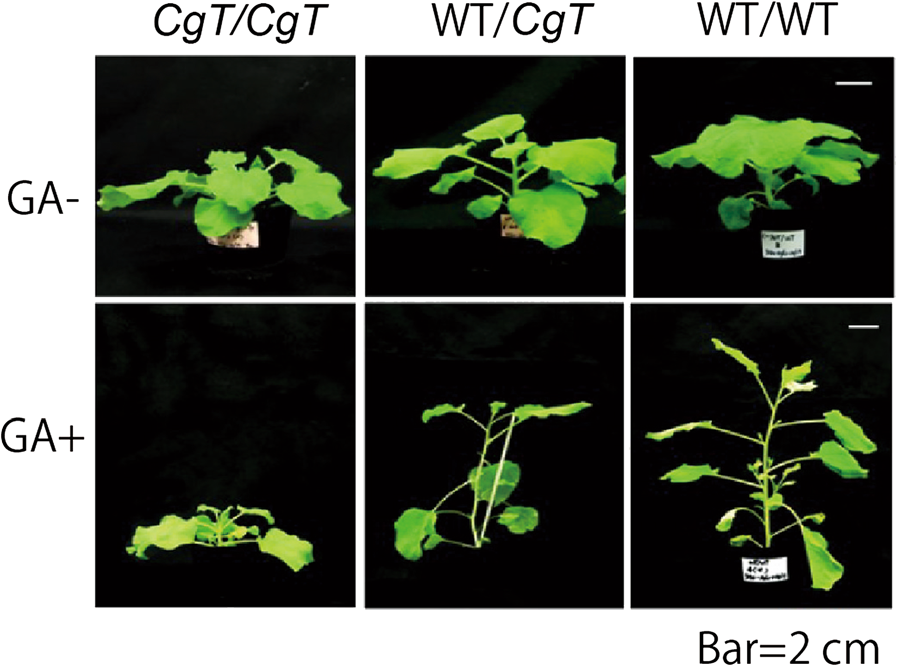
**Shoot phenotypes for different graft combinations.** Upper panels show the plants grown using the respective graft combinations. The lower three show the plants grown after GA_3_ spraying*.*

To determine whether *CgT* rootstock affects growth of a WT scion as a result of *Atgai* mRNA transport, grafts consisting of a WT scion and a *CgT* stock (WT/*CgT*) and a CgT scion and a WT stock (*CgT*/WT) were treated with GA_3_ (Figure 3). Self-grafted WT and *CgT* plants (WT/WT and *CgT/CgT*) were also prepared. These grafted plants were grown on soil in pots and sprayed with water with or without GA_3_. The GA-treated WT/WT and WT/*CgT* combinations showed a typical GA response phenotype: rapid elongation, a slender growth form, and yellowish leaves, but the response of the *CgT*/*CgT* combination was not so obvious (Figure 4). Moreover, the shoot stature of WT/*CgT* was approximately half that of WT/WT, indicating that the WT scion grafted on *CgT* was less sensitive to GA_3_.

**Figure 5:**
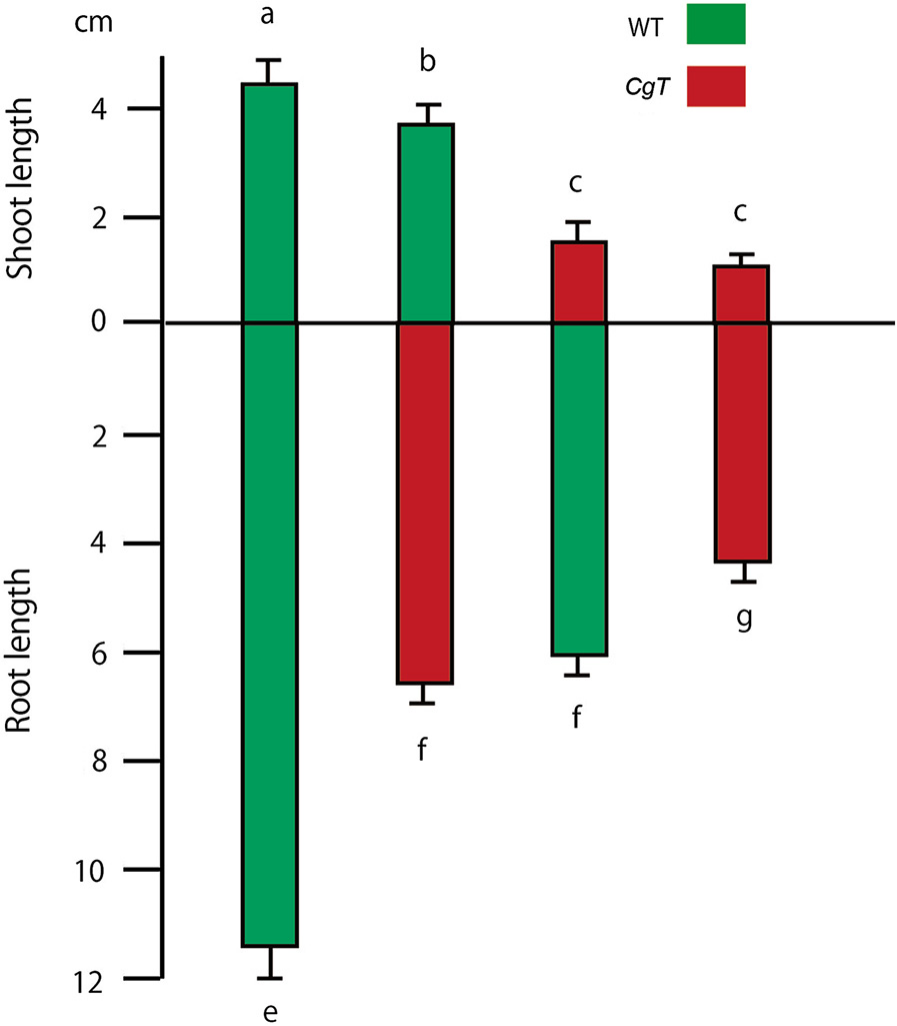
**Average shoot and root lengths of the grafts after GA**_**3 **_**treatment.** Significance of differences was determined by Student’s *t* test with equal or unequal variances as appropriate, and the same letters in each graph indicate that there was no significant difference. After 2 weeks of GA treatment the photographs were taken.

Using hydroponic culture, as shown in Figure 3, the growth rates of the graft shoot and root were measured precisely. The *CgT* rootstock reduced the stature of the WT scion, and the *CgT* scion also reduced the length of the WT rootstock (Additional file 1). To observe the effect of *CgT* on the grafted partner’s mass, shoot and root fresh weights for four graft combinations were measured. The combination showing the highest mass for both the shoot and root was WT/WT, followed in order by WT/*CgT*, *CgT/*WT, and *CgT/CgT* (Figure 5). Histograms were constructed (Additional file 2) using these individual graft data. Although variability among the growth rates of individual grafts was evident, *CgT* obviously reduced the growth rate of the grafted WT scion.

### Transport of *Atgai* mRNA through the graft union

**Figure 6:**
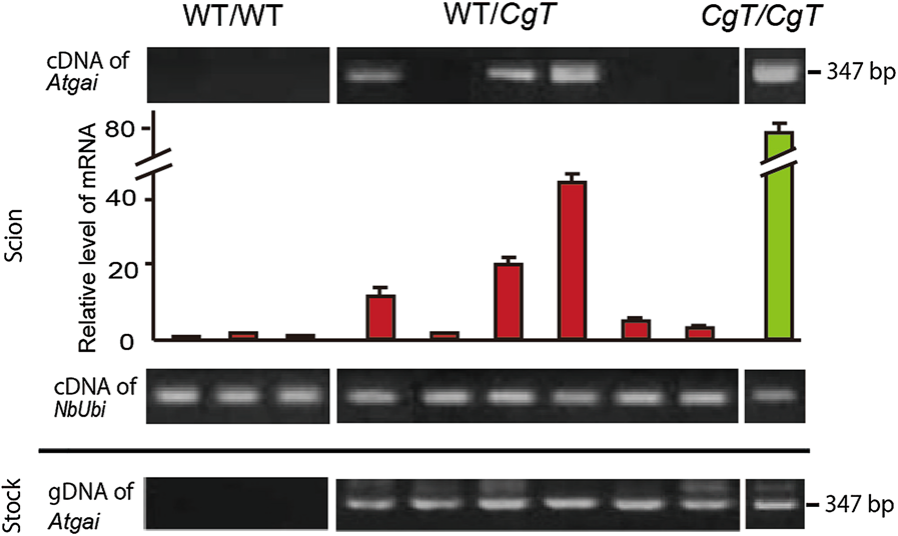
**Detection of *Atgai *mRNA in WT scions on *CgT *rootstock.** At 21 days after grafts had been established, three, six and one were randomly harvested from 21 WT/WT, 63 WT/*CgT*, and 7 *CgT/CgT,* respectively. The scion leaves were used for RT-PCR and qRT-PCR analyses of *Atgai* mRNA. *NbUbi* (ubiquitin of *N. benthamiana*) was amplified as a control. The stock was used for PCR of the *Atgai* transgene.

Any effect of *CgT* rootstock on the WT graft would be caused by long-distance transport of *Atgai* mRNA through the graft union. To confirm this, RT-PCR was used to detect the mutant mRNA in the grafted material. Thirteen WT scions out of 63 WT/*CgT* paired combinations showed amplification of a clear *Atgai* product of the predicted size, 347 bp (Additional file 3), indicating that long-distance transport of *Atgai* transcripts had occurred in some grafts, whereas no product was detected in 21 WT/WT paired combinations. To quantify the transported *Atgai* mRNAs, we chose six WT/*CgT* scions at random and tried to detect the *Atgai* transcript in them using qRT-PCR (Figure 6). Three of the six WT scion samples clearly showed an amplified product; the others showed a very small amount of the amplified product that was detectable only by qRT-PCR. Sequencing of the amplified fragments confirmed that they were derived from *Atgai*, thus demonstrating that transport of the mRNA through the graft unions varied among individuals.

### Detection of Atgai-T7 protein in the scion of the WT/*CgT* combination

**Figure 7:**
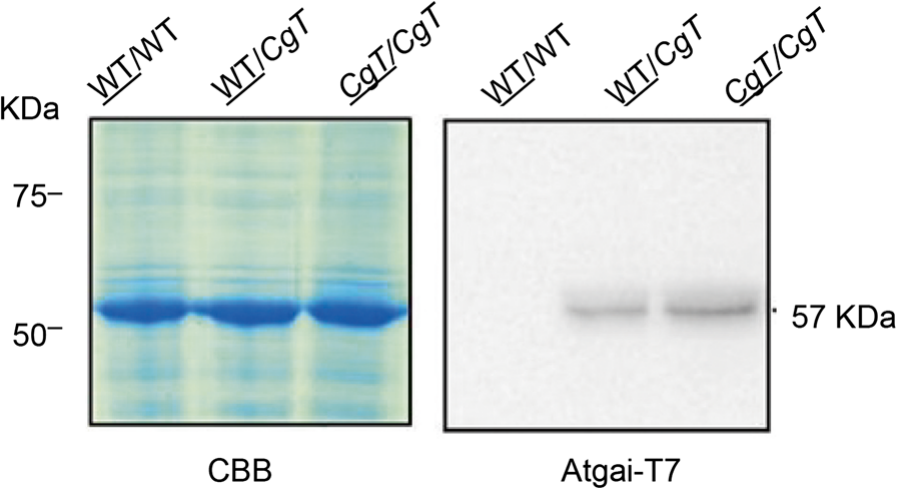
**Western blotting for detection of Atgai-T7 protein in the WT scion grafted onto *CgT *stock.** Using a T7-tag antibody, Atgai-T7 protein was identified in a bulked WT scion sample from five grafts in which *Atgai* mRNA had been positively detected (Figure 5). Bulked samples of WT and *CgT* homogeneous grafts were used as negative and positive controls, respectively. Each lane was loaded with 25 μg protein.

Since *Atgai* mRNA was shown to be transported, we tested whether the mRNA was also present in WT scions grafted onto Δ-DELLA-Atgai rootstock. The scions of WT/WT and *CgT/CgT* homogeneous grafts were used as negative and positive controls (n >5), respectively. Leaves from five scions of the WT/*CgT* combination, in which *Atgai* mRNA had been positively detected, were harvested carefully and analyzed by Western blotting using a T7-tag antibody (Figure 7). A clear band closely matching the predicted size (57 kDa) of Atgai-T7 was detected in the WT scion grafted on the *CgT* stock*.* Although it was considered that Atgai mRNA was transportable from the *CgT* stock to the WT scion and then translated into protein in the scion tissue, the movement of the protein itself from the stock to the scion is also a possibility.

### Attenuation of the GA response of the WT scion on *CgT* stock revealed by microarray

**Figure 8:**
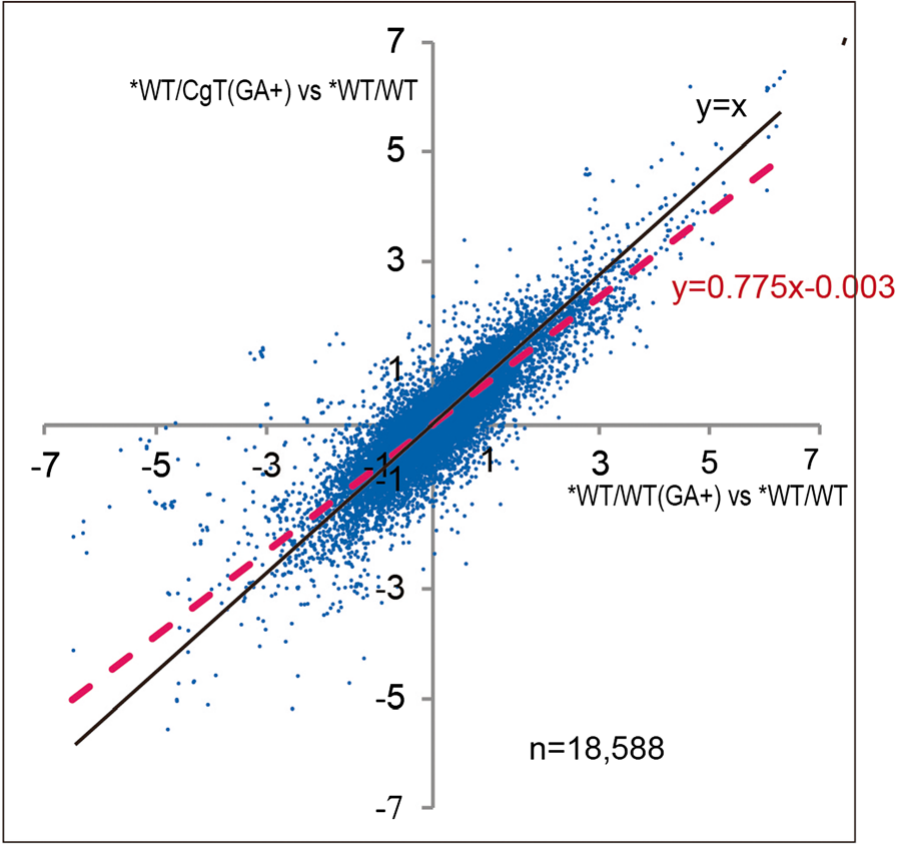
**Relationships among degrees of change in expression of 18,418 genes for different graft combinations.** The X and Y axes represent *WT/WT (GA+) vs *WT/WT and *WT/*CgT* (GA+) vs *WT/WT, respectively. *WT indicates the scions used for microarray analysis. The numerical values were log 2-transformed and the degrees of change were plotted on the respective axes. The calculated regression line is shown as a red broken line.

To investigate in detail the GA response of the scion on *CgT* stock, microarray analyses were performed using three mRNA samples obtained from the scions of the WT/*CgT*(GA), WT/WT(GA), and WT/WT combinations (GA in parenthesis indicates GA_3_-treated plants, as shown in Figure 3), and differences in the resulting changes of gene expression among the *WT/WT (GA) vs *WT/WT and *WT/*CgT* (GA) vs *WT/WT combinations (*WT indicates samples used for RNA extraction) were compared. Out of 18,588 unique genes, genes exhibiting changes in expression of over 100 and below 0.01 in at least one of both combinations were removed. The remaining 18,418 genes were selected, and their changes in expression were plotted on an X-Y scattergram, where the X axis = *WT/WT(GA) vs *WT/WT and the Y-axis = *WT/*CgT* (GA) vs *WT/WT (Figure 8). The regression line from all plots approached the X-axis, with a slope of 0.775, indicating that the GA response of genes in the *WT/*CgT* (GA) combination was weaker than that of genes in the *WT/WT (GA) combination. In order to confirm this, the data from all 18,418 genes were separated into 9 classes according to the degree of the change in expression (Table 1), and the distributions of the numbers of genes were compared between *WT/WT(GA) vs *WT/WT and *WT/*CgT* (GA) vs *WT/WT. The change in expression in the former case showed that 2,115 genes (≥2) and 1,754 genes (≤0.5) exhibited enhanced and reduced expression, respectively. On the other hand, the change in expression in the latter case showed that 1,827 and 1,494 genes exhibited enhanced and reduced expression, respectively. In total, 548 genes (3869–3321) showed a class E change in expression (≤0.5~≥2). These results clearly demonstrated that the WT scions on *CgT* stocks had fewer genes whose expression was altered in response to GA treatment, resulting that the *CgT* rootstock attenuated the GA response in the WT scion.

**Table 1:** Numbers of genes exhibiting different degrees of GA response

**Class (degree of change)**	**Numbers of genes**	
	*WT/WT(GA) to *WT/WT	*WT/ *CgT( * GA) to *WT/WT
A (<20 ~ ≤100)	35	24
B (<10 ~ ≤20)	81	45
C (<5 ~ ≤10)	331	182
D (<2 ~ ≤5)	1668	1576
E (<0.5 ~ ≤2)	14549	15097
F (>0.5 ~ ≥0.4)	786	663
G (>0.4 ~ ≥0.3)	493	412
H (>0.3 ~ ≥0.2)	248	262
I (>0.2 ~ ≥0.01)	227	157

### *Atgai* apple rootstock reduces the stature of the scion cultivar

**Figure 9:**
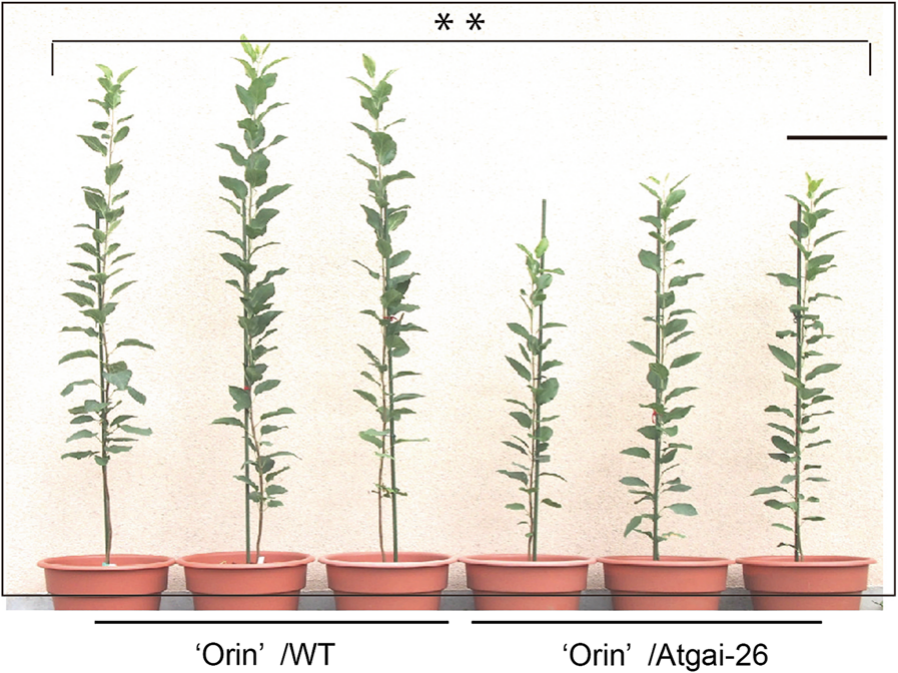
**Plants produced by grafting scions of the *Malus *cultivar 'Orin’ onto wild-type *M. prunifolia *or *Atgai *transgenic (Atgai-26) rootstock.** Grafting was performed at the shoot culture stage, and the rooted plants were then grown for 15 months through one winter dormancy. **P<0.01 (Student’s *t* test) for comparisons between the two graft combinations.

*Malus prunifolia* is a non-dwarf-type apple rootstock used predominantly in Japan. We attempted to transform *M. prunifolia* by introducing the *Atgai* gene by the *Agrobacterium* method. Several putative transgenic lines were obtained in two transformation experiments. Through propagation of the shoots on a medium containing the selection marker, only one line (Atgai-26) was confirmed as transgenic by Southern blot hybridization (Additional file 4). The limitations of obtaining a single transgenic line was likely due to the low transformation efficiency in *Malus* species and the effect of the *Atgai* introduced, suppression of GA signaling. The Atgai-26 *M. prunifolia* exhibited a semi-dwarf phenotype with reduced sensitivity to GA treatment, as is the case for *CgT* tobacco (Additional file 5). Moreover, the grafts between Atgai-26 stock and scions of the apple variety 'Orin’ showed a clearly reduced shoot stature (Figure 9). Although these results are based on a single transgenic apple line, the data are consistent with the results in tobacco.

## Discussion

Lack of the DELLA domain of GAI results in constitutive activation of a mutant growth inhibitor whose genetically dominant action can no longer be opposed by GA. Since *gai* is a semi-dominant and gain-of-function mutation [8], integration of *gai* into WT results in a semi-dwarf phenotype, and this has contributed to the Green Revolution [29]. The stature of several crops, including rice [13], *Chrysanthemum*[30], tobacco [27], and apple [28], has been reduced by transformation with *ΔDELLA-GAI* (*gai*), as is the case for the *Arabidopsis gai-1* allele. In all cases, *ΔDELLA-GAI* (*gai*) was expressed under the control of *CaMV 35S* (*Cauliflower Mosaic Virus* promoter 35S).

On the other hand, *GAI* mRNA is able to move through phloem, and is considered to function at the site(s) to which it has been transported [14,18]. As plasmodesmata connect the functional enucleate sieve elements of the phloem to their neighboring companion cells [31], this pathway is considered to allow the selective entry of information macromolecules into the phloem translocation stream. In this study, the *Atgai* transgene was delivered via the promoter of a plant virus, CoYMV, which is expressed strongly only in companion cells [32]. Intriguingly, the *CgT* transgenic tobacco plants also exhibited semi-dwarf phenotypes, as was the case when the 35S promoter was used, indicating that expression of *Atgai* only in companion cells can induce a definite dwarf phenotype. Therefore, it is suggested that the *GAI* transcript in companion cells acts non-cell-autonomously to regulate the growth of plants. Furthermore, the less sensitive GA response of the WT scion on *CgT* rootstock strongly indicated the non-cell-autonomous effect of the *GAI* transcript. In higher plants the system of *GAI* mRNA transport through the phloem might function to integrate growth modalities among plant organs [14]. In our experiments, not only the integrated *Atgai* but also the extant *N. benthamiana GAI* were considered to be transported by the same molecular mechanism. Detailed analysis of the transport of both gene transcripts via the graft union would be expected to clarify the physiological role of *GAI* mRNA long-distance movement.

Approximately one-fourth of the grafts we examined did not show the RT-PCR product derived from the *gai* mRNA transported from the stock. Long-distance transport of RNA in sieve tubes appears to be mediated by RNA-binding proteins. Ham et al. [17] identified RNA-binding proteins involved in mRNA transport, and proposed a model in which a ribonucleoprotein complex moves in the phloem. It is clear that RNA also binds to chaperone proteins for stability and delivery to target tissues [18]. As a matter of course, such large complexes must pass through the graft union, where vascular bundles are developed in the callus (Additional file 6). *De novo* passage through the sieve tube tends to be unorthodox, showing features such as a winding path, thus disrupting the passage of large ribonucleoprotein complexes, and in extreme cases such complexes would become clogged. Since the conductance of a vessel is proportional to the fourth power of the vessel radius (Hagen-Poiseulle law), a slightly reduced diameter would pose an obstacle to passage. On the other hand, grafting of many horticultural crops is a well-developed technology, and this phenomenon in such plants might be less problematic, suggesting that they can establish near-complete connection of the sieve tube at the graft union as the growth progressed.

No other report has identified the translated protein derived from the transported *gai* mRNA in grafting experiments. In the WT scion on the *CgT* stock, the *gai* protein fused with the T7-tag peptide was clearly detectable. Its amount was considered to be approximately one-third of that in the *CgT* stock, suggesting effective translation of the *gai* mRNA in the scion. However, there is a possibility that the protein might have been translated in companion cells and then moved through the graft union. For clarification of this issue, a high-accuracy experiment to detect the GAI protein in sieve elements would be necessary.

Our present microarray experiment was also the first of its kind to investigate the long-distant distance transport of mRNA in grafted plants. The overall results clearly revealed that the WT scions on *CgT* stocks had fewer genes whose expression was altered in response to GA treatment than in the WT scions on WT stocks. It is known that GA has pronounced effects on overall reproductive growth from flowering to fruiting [33]. Therefore, although we succeeded in growing a dwarf apple tree using the *Atgai* rootstock, the effect of this manipulation on subsequent fruit development will require careful investigation. However, semi-dwarf cultivation of many crops might be feasible using *Atgai-*expressing rootstock. Furthermore, the application of not only *GAI* but also other phloem-transportable mRNAs [21,23,26] might become possible by means of grafting. Finally, the present results imply that a non-genetically modified scion is capable of improvement by a genetically modified rootstock, and consequently, the fruits of the plant would not contain the inserted DNA sequence. Grafting using gene-modified rootstock is expected to become a focus of interest as an innovative approach to agriculture [34].

## Conclusions

This study has demonstrated for the first time that transgenic stock expressing a ΔDELLA-*gai* gene by the companion cells specific-promoter is able to transfer the *gai*-mRNA to a wild-type scion. Furthermore, this study provides the first evidence that the translated product of the *gai* mRNA is present in the scion. In addition, microarray data clearly indicated that many GA-responsive genes in the wild-type scion on ΔDELLA-*gai* stock show attenuated responses.

## Methods

### Plant materials and growth conditions

*Nicotiana benthamiana* was grown on MS [35] agar (Wako Pure Chemical Industries Ltd., Osaka, Japan) plates in a Petri dish or a glass bottle. Grafted tobacco plants were cultivated on soil in a pod. Hydroponic culture was also performed using a styrofoam plate floating on hydroponic solution (Otsuka House Nos. 1 and 2, Otsuka Chemical Co., Osaka, Japan). The cultures were incubated at 24°C with a 16:8-h photoperiod under 100 μmol m^-2^ s^-1^ provided by cool-white fluorescent tubes. Wild apple rootstock (*Malus prunifolia* Borkh. var. *ringo* Asami Mo 84-A) was used for transformation. The grafts of *Malus* plants were transplanted on a pod and grown in a greenhouse [36].

### Construction of the binary vector

*Arabidopsis* seed (*gai*-1, CS63) was provided by the *Arabidopsis* Biological Resource Center (Ohio State University). The CS63 plantlets were used to extract the RNA fraction to obtain the full cDNA of the *Atgai* gene harboring a 51-bp deletion from the region encoding the conserved DELLA domain [9]. *Xba*I and *Sac*I restriction sites were added to the 5′- and 3′- sites of the *Atgai* fragment by PCR using the primers AtgaiXba and AtgaiSac (Additional file 7). The GUS fragment was deleted from *pBI121* with *Xba*I and *Sac*I [37], and then replaced with the new *Atgai* fragment. The 35S promoter was deleted from the vector by *Sal*I and double-digested by with *Spe*I and *Xba*I. The *Commelina yellow mottle virus* promoter (*CoYMVp*) of pCOI [38] (from Prof. Neil Olszewski, University of Minnesota, St. Paul, MN, USA), which is expressed specifically in companion cells [39], was amplified by the primers CoYMVproFPSal and CoYMVproRPSpe, and then used to replaced the 35S promoter sequence with the *Sal*I and *Spe*I sites (Additional file 7). A T7-epitope tag sequence (MASMTGGQQMG, Invitrogen, USA) was inserted into the 3′- site of *CoYMVp: Atgai* by PCR. The forward primer was CoYMVproFl, and the primer T7tag R including the T7-epitope tag sequence was used as the reverse primer. The new vector was sequenced to ensure correct insertion. The *CoYMVp:Atgai-T7* (*CgT*) fused gene was cloned in *pBI121* (Figure 1) carrying the nos-kanamycin resistance cassette. Transgenic lines were identified by PCR with the primers CoYMproFP1 and AtgaiR1 to detect the Δ-DELLA –gai. kihyufg (Additional file 7).

### Grafting experiment

Micrografting was performed according to the method described by Bai et al. [40]. Briefly, as shown in Figure 2, plantlets 10 days after germination were grafted under a stereomicroscope on a clean bench. The plant was cut horizontally approximately 3 mm below the cotyledon. Then the scion (tissue with the cotyledon) and rootstock (tissue at the bottom with root) were fastened together with a silicone tube (ф 0.4 mm × 0.1 mm, 3 mm length; TechJam, Osaka, Japan). The grafted plants were each propped against an agar block on MS agar medium. At 7 days after grafting, the silicone tube was cut off. The grafts were then cultivated in soil or using a standard nutrient solution (Otsuka House Nos. 1 and 2, Otsuka Chemical Co., Osaka, Japan) until phenotype observation or sampling. In the case of *Malus* plants, cleft grafting between subcultured shoots was performed.

### GA_3_ treatment

Grafted plants that had been grown for 7 days in a Petri dish were transferred to pots with nursery soil. After 1 week, they were sprayed with 0.1 mM GA_3_ solution (Nakarai Tesque, Inc. Kyoto, Japan) containing 0.02% Tween 20 once every two days for three weeks. For RT-PCR, protein extraction, microarray and seedling stature measurement, plants were cultured hydroponically for 9 days, then sprayed with 0.1 mM GA_3_ solution_._

### RNA extraction, RT-PCR and qRT-PCR analysis

Total RNA was extracted from leaves with TRIzol reagent (Invitrogen, Tokyo, Japan), and genomic DNA was eliminated with a TURBO DNA-free Kit (Ambion Inc., Austin, USA). Reverse-transcribed cDNAs were prepared using a SuperScript® VILO™ cDNA Synthesis Kit (Invitrogen, USA). The cDNA corresponding to 50 ng of total RNA was used in 10-μl reactions with an S1000 Thermal Cycler (Bio-Rad, USA). To amplify *Atgai* mRNA in the WT scion, primers (AtgaiF2 and AtgaiR2) and the nested primers (AtgaiF3 and AtgaiR3) were prepared. The amplification condition were as follows: initial denaturing at 94°C for 4 min, 25 cycles at 94°C for 30 s, 58°C for 30 s, and 72°C for 1 min; and extension at 72°C for 3 min. For the nested PCR, 1 μl of the first PCR product was used as the template and subjected to 30 cycles. For qRT-PCR, 1 μl of the cDNA corresponding to 50 ng of total RNA was used in 20-μl reactions with iQ SYBR Green Supermix (Bio-Rad, USA). Triplicate reactions for each sample were amplified along with non-template controls on a Chromo 4 real-time PCR detector (Bio-Rad, USA). *NbUbi* (Accession No.: AY912494) was used to normalize the expression levels of *Atgai*. Primers (Atgai QF/Atgai QR and Ubi QF/ Ubi QR) specific to the *Atgai* and *NbUbi* genes were used in this experiment (Additional file 7).

### Protein extraction and immunoblotting

About 3.0 g of scion shoot tissue (n = 5) was sampled after mRNA transport had been positive detected, and then ground in liquid nitrogen using a pre-cooled mortar and pestle. Protein was extracted according to the method described previously [41]. The concentration of protein was measured by DC Protein Assay (Bio-Rad, USA). Total proteins (25 μg) were mixed with a SDS loading buffer, and heated at 95°C for 5 minutes for denaturation, then fractionated by SDS-PAGE (sodium dodecyl sulfate polyacrylamide gel electrophoresis). SDS-PAGE was performed in 12.5% polyacrylamide gels using Bio-Rad Mini-PROTEAN 4 equipment at 200V for 1 h. Proteins were transferred to Immun-Blot™ PVDF membranes (Bio-Rad, USA). Then, each membrane was blocked with BSA (bovine serum albumin, Sigma, Germany) at room temperature for 1 h (in the blocking buffer, BSA was dissolved in 1 × TBS- 0.1% Tween to a final concentration 0.02 g/mL). For analysis of the immunoblots, the membranes were incubated with 0.1 μg/mL anti-T7-peptide monoclonal antibody (Novagen, USA) at 4°C overnight, and then washed 4 times for 1 h at room temperature. The membranes were then incubated with a 2,000-fold dilution of anti-mouse IgG, Goat Poly HRP (Cosmo Bio Co., Ltd., Tokyo, Japan) for 1 h, and washed 3 times for 1 h at room temperature. The signals were detected using an Amersham ECL Plus Western Blot Detection System (GE Healthcare, UK). A duplicate gel was run at the same time and then stained with Coomassie Brilliant Blue R250 as a loading control.

### Microarray analysis

Five WT scions in which the *Atgai* mRNA transported from the *CgT* stock had been detected by RT-PCR were combined, and total RNA was prepared from the sample. Five scions for the WT/WT combination with and without GA_3_ treatment were also combined and used as samples for RNA extraction. The purification, labeling of cRNA, hybridization to 44K Tobacco DNA microarray (Agilent Techologies), signal scanning, and processing were performed by Hokkaido System Science Co., Ltd. (Japan) with a Low Input Quick Amp Labeling Kit using an Agilent Technologies Microarray Scanner (Agilent Techologies, USA). A total of 18,588 unique genes that passed the stringent quality control were used for inspection.

## Abbreviations

CgT: CoYMVp:Atgai-T7; GA: Gibberellic acid; GAI: Gibberellic acid insensitive; MS: Murashige and Skoog medium; T7: T7 (bacteriophage) -epitope tag; CoYMVp: *Commelina yellow mottle virus.*

## Competing interests

The authors declare that they have no competing interests.

## Authors’ contribution

HX and TH designed the experiments; HX and RI conducted most of research and analyzed the data together with TL and TH; all authors contributed to the writing of the manuscript. All authors read and approved the final manuscript.

## Supplementary Material

Additional file 1 **Shoot and root growth rates in respective grafts after GA**_**3 **_**treatment.** Click here for file

Additional file 2 **Histograms of shoot and root lengths of grafts between different graft combinations.** The arrowhead indicates the average of the population. Click here for file

Additional file 3 **RT-PCR detection of *CgT *** **transcript of grafts between different graft combinations.** Click here for file

Additional file 4 **Southern blot result of Atgai-26 with *Atgai *probe.** 15 μg of gDNAs were loaded in each lane. Lane 1: digested by *Eco*RV. Lane 2: digested by *Hin*dIII. +: plasmid as positive control. Click here for file

Additional file 5 **Stature of Atgai-26 and Wild Type *M. prunifolia.*** At four weeks after spraying the water with or without GA_3_ (0.1 mM), the shoot statures were photographed. Click here for file

Additional file 6 **Turbulent structure of phloem line at graft junction of *N. benthamiana.*** The wild type plant were grafted on CoYMV:GUS transgenic plant by micro-grafting technique. After 2 weeks, the phloem conflation was observed by GUS staining. Click here for file

Additional file 7 Primers sequences used in this study. Click here for file
