# Supplementary material for: Long-distance transport of Gibberellic Acid Insensitive mRNA in Nicotiana benthamiana
Source: BMC Plant Biol. 2013 Oct 21;13:165. doi: 10.1186/1471-2229-13-165 (PMC4015358; doi:10.1186/1471-2229-13-165)
Supplement: Additional file 1 — Shoot and root growth rates in respective grafts after GA 3 treatment. [file 1471-2229-13-165-S1.pdf]

Additional file 1. Shoot and root growth rates in respective grafts after GA_3 treatment.

Grafting pattern (scion / stock)	Length (cm)		Flesh weight (mg)	
	Shoot	Root	Shoot	Root
WT / WT	4.13±0.16	10.47±0.48	490.0±27.1	55.8±3.7
WT / *CgT*	3.41 ± 0.14*	6.01 ± 0.24**	314.5 ± 23.9**	30.9 ± 3.3**
CgT / WT	1.52 ± 0.19**	5.60 ± 0.34**	214.3 ± 25.0**	23.4 ± 2.4**
CgT / CgT	1.03 ± 0.19**	3.98 ± 0.42**	145.1 ± 18.8**	10.9 ± 1.8**

Values are expressed as mean ± SE. Significant differences from WT/WT were determined by Student's *t* test with equal or unequal variances as appropriate (*$P < 0.01$ or **$P < 0.001$).
