# Supplementary material for: Long-distance transport of Gibberellic Acid Insensitive mRNA in Nicotiana benthamiana
Source: BMC Plant Biol. 2013 Oct 21;13:165. doi: 10.1186/1471-2229-13-165 (PMC4015358; doi:10.1186/1471-2229-13-165)
Supplement: Additional file 2 — Histograms of shoot and root lengths of grafts between different graft combinations. The arrowhead indicates the average of the population. [file 1471-2229-13-165-S2.pdf]

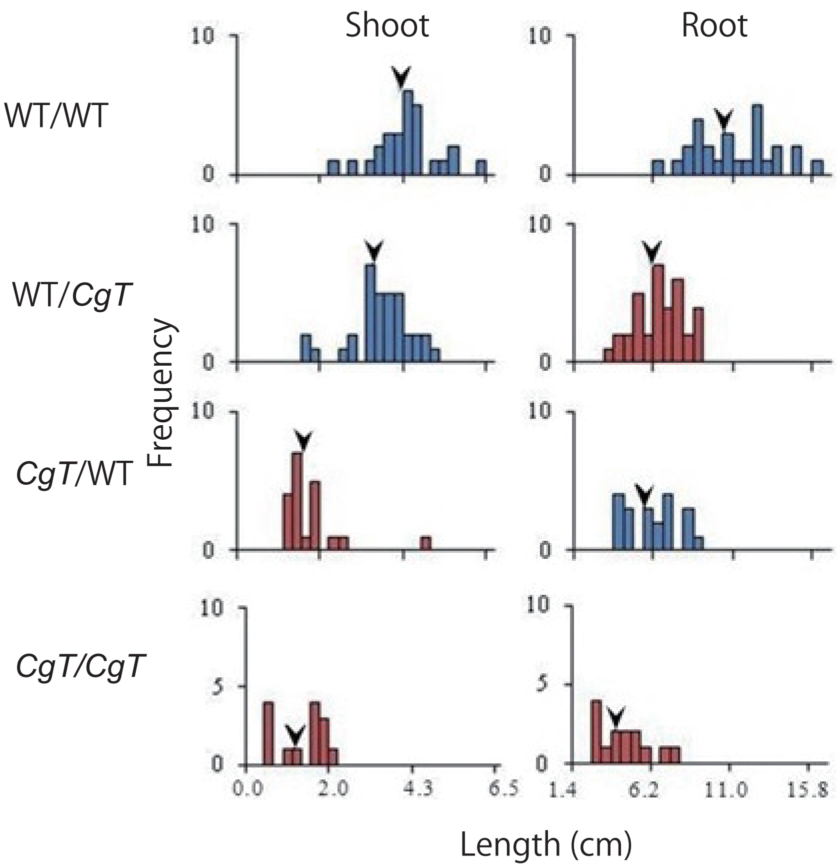

Additional file 2. Histograms of shoot and root lengths of grafts between different scion and stock combination. The arrowhead indicates the average of the population.
