# Supplementary material for: Long-distance transport of Gibberellic Acid Insensitive mRNA in Nicotiana benthamiana
Source: BMC Plant Biol. 2013 Oct 21;13:165. doi: 10.1186/1471-2229-13-165 (PMC4015358; doi:10.1186/1471-2229-13-165)
Supplement: Additional file 3 — RT-PCR detection of CgT transcript of grafts between different graft combinations. [file 1471-2229-13-165-S3.pdf]

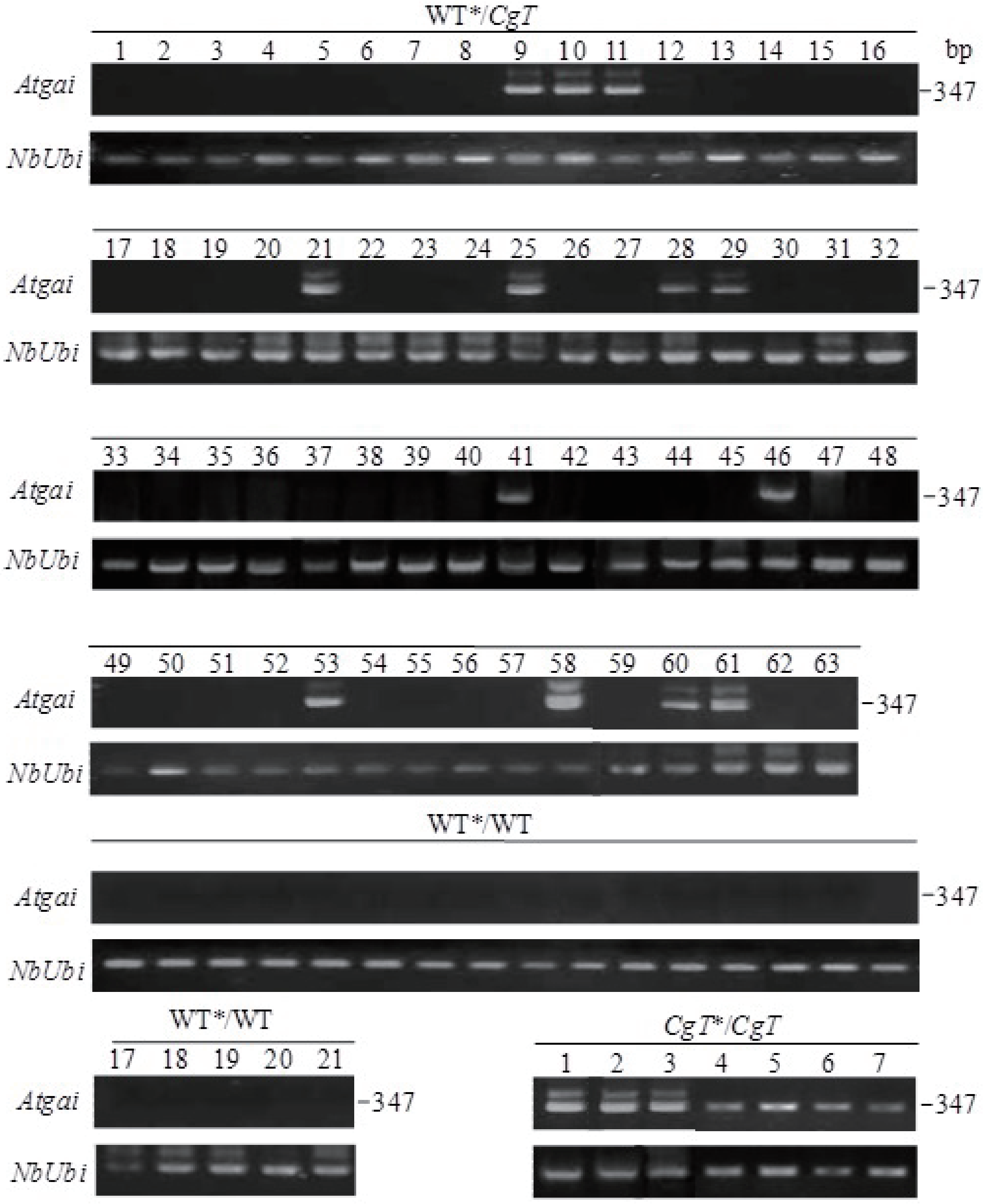

Additional file 3 RT-PCR detection of *CgT* transcript of grafts. between different graft combinations. Astriscs mean samples used for the detection.
