# Supplementary material for: Long-distance transport of Gibberellic Acid Insensitive mRNA in Nicotiana benthamiana
Source: BMC Plant Biol. 2013 Oct 21;13:165. doi: 10.1186/1471-2229-13-165 (PMC4015358; doi:10.1186/1471-2229-13-165)
Supplement: Additional file 4 — Southern blot result of Atgai-26 with Atgai probe. 15 μg of gDNAs were loaded in each lane. Lane 1: digested by EcoRV. Lane 2: digested by HindIII. +: plasmid as positive control. [file 1471-2229-13-165-S4.pdf]

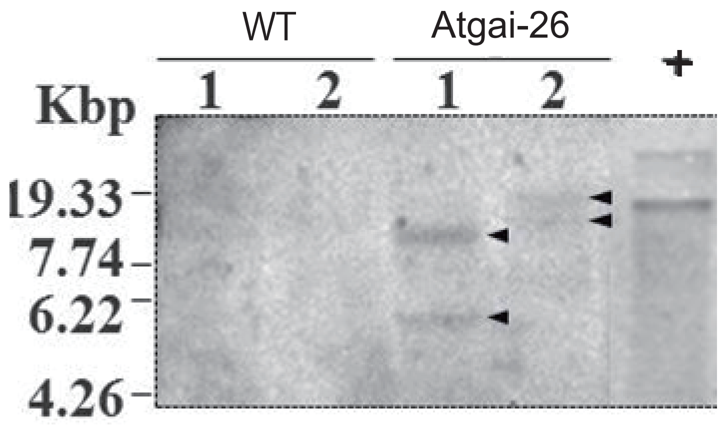

Additional file 4 Southern blot result of wild type and Atgai-26 plants with *Atgai* probe. 15 µg of gDNAs were loaded in each lane. Lane 1: digested by *Eco*RV. Lane 2: digested by *Hin*dIII. +: plasmid as positive control.
