# Supplementary material for: Long-distance transport of Gibberellic Acid Insensitive mRNA in Nicotiana benthamiana
Source: BMC Plant Biol. 2013 Oct 21;13:165. doi: 10.1186/1471-2229-13-165 (PMC4015358; doi:10.1186/1471-2229-13-165)
Supplement: Additional file 5 — Stature of Atgai-26 and Wild Type M. prunifolia. At four weeks after spraying the water with or without GA3 (0.1 mM), the shoot statures were photographed. [file 1471-2229-13-165-S5.pdf]

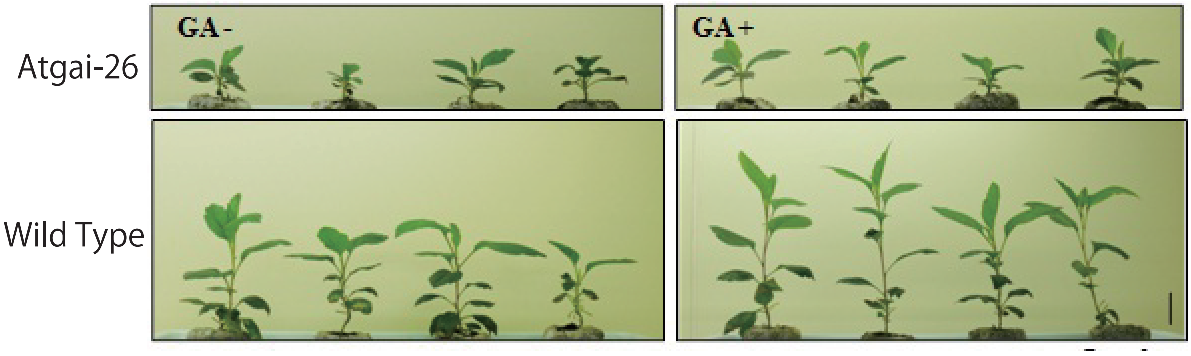

Additional file 5 Stature increase of Atgai-26 and Wild Type *M. prunifolia* by GA_3 treatment. At four weeks after fourth GA_3 treatment (once every two days), the shoot statures were photographed .
