# Supplementary material for: Long-distance transport of Gibberellic Acid Insensitive mRNA in Nicotiana benthamiana
Source: BMC Plant Biol. 2013 Oct 21;13:165. doi: 10.1186/1471-2229-13-165 (PMC4015358; doi:10.1186/1471-2229-13-165)
Supplement: Additional file 6 — Turbulent structure of phloem line at graft junction of N. benthamiana. The wild type plant were grafted on CoYMV:GUS transgenic plant by micro-grafting technique. After 2 weeks, the phloem conflation was observed by GUS staining. [file 1471-2229-13-165-S6.pdf]

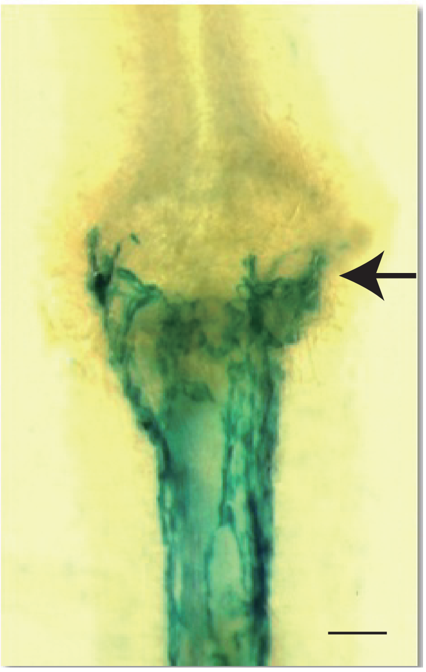

Additinal file 6 Turbulent structure of phloem line at graft junction of ***N. benthamiana***. The wild type plant were grafted on CoYMV:GUS transgenic plant by micro-grafting technique. After 2 weeks, the phloem confugation was observed by GUS staining.
Bar indicates 1 mm.
