# Supplementary material for: Long-distance transport of Gibberellic Acid Insensitive mRNA in Nicotiana benthamiana
Source: BMC Plant Biol. 2013 Oct 21;13:165. doi: 10.1186/1471-2229-13-165 (PMC4015358; doi:10.1186/1471-2229-13-165)
Supplement: Additional file 7 — Primers sequences used in this study. [file 1471-2229-13-165-S7.pdf]

Additional file 7 Primers list.

Name of primers	Sequences
Atgai Xba	5′- GCTCTAGA ATGAAGAGAGATCATCATCATCAT -3′
Atgai Sac	5′- GCGAGCTCAGTAATTTAGGCGAGATTAAAAT -3′
CoYMV pro FP Sal	5′- GCGTCGACGGTATCGATTTCTTAGG -3′
CoYMV pro RP Spe	5′- GCACTAGTAGTAATTTAGGCGAGATTAAAAT -3′
T7 tag R	5′- AGCGGTACCCTATCCCATTTGTTGTCCTCCAGTCAT AGAAGCCATTCCTCCATTGGTGGAGAGTTT -3′
CoYMV pro F1	5′- CCTATGCCTTTATTCGCAGC -3′
Atgai R1	5′- TCAAGCCACGTGTAAAGCTCCGC -3′
Atgai F2	5′- TTGAGCTTAGACCAAGTGAGATTG -3′
Atgai R2	5′- CTGACTCAACGTTTCATGACGCTCA-3′
Atgai F3	5′- TCGGAGCTATGAGACAAGTCG-3′
Atgai R3	5′-ACGTGAATCGCCTCAGCTAAA-3′
Atgai QF	5′- CAACTCGGCATGTTGTCCTGGTTG -3′
Atgai QR	5′- TTCGGCGAAGTAAGTAGCGAC -3′
Ubi QF1	5′- CAGGACAAGGAGGGTATC -3′
Ubi QR1	5′- CACGTCATCAACAACAGA -3′
